# Medico-Social Questions and the War: The Profession and the Politicians

**Published:** 1915-02-20

**Authors:** 


### MEDICO-SOCIAL QUESTIONS AND THE WAR.

### The Profession and the Politicians.

The chapter of events has pushed medical activities into great public prominence, and certainly the profession has little reason to complain of the tone of the discussion. It might be possible to quote some very different experiences, but no one wishes to revive controversies which a national peril has pushed into the background. With Parliament in session, a debate on anti-typhoid inoculation was, of course, inevitable. It produced the familiar facts and figures, and there was no really serious attempt to controvert them. What really occupied the members were the arguments for and against compulsion. Strictly speaking, this is a question not of medical science, but of public policy. The profession, as was its duty, has collected the facts and has submitted them to the Army authorities. On the other hand, it would appear that a certain number of the new soldiers object to inoculation, and the suggestion is made that some men may be deterred from joining the ranks if compulsion is enforced. What the authorities have to do is to balance one of these considerations against the other.

### Clouding the Issues.

It surely seems a strange thing that the value of the practice cannot be discussed apart from exaggeration and passion. To say, as was said in the recent debate in the House of Commons, that someone had an interest in the suppression of the truth, is to make reasonable discussion impossible. Equally absurd is it to suggest that the non-adoption of compulsion is a reflection on the capacity of the medical profession. Whether inoculation is or is not a valuable agency in the prevention of enteric fever is a question that ought to be discussed in the same temper as would attend the consideration of the value of any other preventive measure. Unfortuately, in some minds the problem is entangled with other questions which have long been highly controversial, while to others the refusal of inoculation appears anti-patriotic. In the meanwhile the practice remains on the voluntary basis, and this being so, the methods of persuasion had better be left in the hands of suitable persons and not delivered as commands in the tones of the drill-sergeant.

The value of alcohol to troops subjected to the conditions of trench warfare is another question in which medical opinion is involved. No one seems to doubt that, for the armies now in training, total abstinence is the best and safest policy, and this is certainly a great advance on the teaching of, say, thirty years ago. The point of controversy is the ration of rum which in certain circumstances may be served out to soldiers on active service. Here, at all events, the issue is not attended with any question of compulsion, for no one suggests that men must take the rum if they object to do so. Letters from individual combatants are quoted both on one side and the other, and no real help comes from the usual platform denunciations of the evils of excessive drinking. Nor is it to the point to say that hot soup, tea, and so on would be more valuable than rum, seeing that in many cases there is no possibility of these beverages. What has to be determined is whether a small dose of rum is or is not of value to a man who has been exposed to cold and wet and who is more or less exhausted both mentally and physically. That it entails all the evils prophesied of it by certain extremists is hard to believe, and, seeing that the supreme desire of the military authorities is to keep their men as effective as possible, most people will be content to let the settlement of the question rest with the soldiers.

### A Fruitless Debate.

Medical discussion has recently been applied in still another direction—namely, to the relative fighting value of the tall and the short man—but it cannot be said we are much further forward for the various contributions which have appeared. The tall man certainly was the more fashionable in early Prussian days, when Europe was ransacked to secure specimens for Friedrich Wilhelm's pet regiment. But now we are told that while a tall soldier is more imposing and dignified in time of peace, this experience may establish a bias in his favour which may be misleading in judging of him as a fighting unit. Not only so, but it seems that he has longer legs than his shorter comrade, in which remark there may be detected perhaps a subtle suggestion that he is the more ready to run away. The shorter man, on the other hand, though less gifted with length of limb, often has the "essential organs of life" more satisfactorily developed. Yet all this reasoning does not lead to a confident vote for the "short-legged, short-necked, short-tempered man" reported as the choice of a Highland doctor. The conclusion of the lecturer is merely that no one can dogmatise upon the question whether tall or short men make the better soldiers.

Once again, therefore, the fact remains that, in spite of eloquence and print, we are left exactly where we were before the debate began. Mr. Kipling says that the most useful thing a civilian can do in these busy days is to speak as little as possible. On this point it may be doubted whether he has made many disciples. So far his speech on the physiological action of music has escaped the attention of the medico-psychologists, and the valuable or other influences of bands on marching troops has yet to undergo analysis. Last of all is the large discussion of war as a factor in biology. On the one side is the argument that, as strength comes from struggle, a nation which never fights must necessarily become deficient in virility and in the capacity to survive. On the other, we learn that as in war the strong and brave are slain while the cowards and weaklings abide, the succeeding generations will inevitably reproduce the characters of the latter, with resulting national deterioration. This is, indeed, to leave us a painful choice, for the prospects are unwelcome whichever horn of the dilemma we select. Is it a weak compromise to suggest that not all the qualities bred in man by war are unhandsome or unhealthy ones, and that without it he might have been a less heroic figure than he is to-day, while at the same time allowing that because an influence has been advantageous at an early stage of evolution it by no means follows that it is to be continued indefinitely? But whatever advice medical or other experts may give on the subject, the world in all probability will continue to take its own way. Men have plenty of opportunity for struggle without warfare, and the heroic virtues are as much displayed in contests with Nature in the Tropics or the Arctic as on the battlefield. Did not Stanley's autobiography show he was made quite as much a man by the early days in the workhouse as by the later years of exploration? Classes may get obese by prosperity, but, when all is said, a high level of moral courage is worth more than a sporadic heroism in emergencies that may happen only once in a lifetime, if they ever happen at all.

